# Inverse Agonist of Retinoid-Related Orphan Receptor-Alpha Prevents Apoptosis and Degeneration in Nucleus Pulposus Cells via Upregulation of YAP

**DOI:** 10.1155/2021/9954909

**Published:** 2021-07-28

**Authors:** Tongzhou Liang, Jincheng Qiu, Shaoguang Li, Zhihuai Deng, Xianjian Qiu, Wenjun Hu, Pengfei Li, Taiqiu Chen, Zhancheng Liang, Hang Zhou, Bo Gao, Dongsheng Huang, Anjing Liang, Wenjie Gao

**Affiliations:** Department of Orthopedics, Sun Yat-sen Memorial Hospital of Sun Yat-sen University, 107 West Yan Jiang Road, Guangzhou, Guangdong 510120, China

## Abstract

Intervertebral disc degenerative disease (IDD) is the most common degenerative spine disease, which leads to chronic low back pain and symptoms in the lower extremities. In this study, we found that ROR*α*, a member of the retinoid-related orphan receptor family, is significantly elevated in nucleus pulposus tissue in IDD patients. The elevation of ROR*α* is associated with increased apoptosis of nucleus pulposus (NP) cells. Therefore, we applicated a well-established inverse agonist of ROR*α*, SR3335, to investigate its role in regulating NP cell metabolism and apoptosis. To further investigate the mechanism that SR3335 regulates the pathogenesis of IDD *in vitro*, tumor necrosis factor alpha (TNF-*α*) stimulation was used in human NP cells to mimic the hostile environment that leads to degeneration. We found that SR3335 treatment reversed the trend of increased apoptosis in NP cells induced by TNF-*α* treatment. Next, TNF-*α* treatment upregulated the expression of type II collagen and aggrecan and downregulated MMP13 (matrix-degrading enzyme matrix metalloproteinase 13) and ADAMTS4 (a disintegrin and metalloproteinase with thrombospondin motifs 4). However, these effects were reversed after SR3335 treatment. Furthermore, we find that SR3335 mediated the effect in NP cells by regulating the YAP signaling pathway, especially by affecting the phosphorylation state of YAP. In conclusion, the reduction of matrix degradation enzymes and apoptosis upon SR3335 treatment suggests that SR3335 is a promising drug in reversing the deleterious microenvironment in IDD patients.

## 1. Introduction

Intervertebral disc degenerative disease (IDD) is one of the most common degenerative spine diseases that cause low back pain and subsequent chronic disability in the elderly [1]. Because of the high prevalence and lack of effective treatment, IDD imposed a huge social and medical care system burden [2]. However, due to incomplete understanding of the etiology of IDD, the treatment now can only provide pain relief during the exacerbation phase, and many patients will eventually have to receive surgery despite these pharmacological therapies [3]. Therefore, it is greatly needed to explore the pathogenesis of IDD and develop new targets for treating IDD. Although the etiology of IDD remains unclear, some common features are suggested to be involved in the development of IDD, including biomechanics alternation, nucleus pulposus (NP) cell apoptosis, and loss of extracellular matrix (ECM) components [4]. During the progression of IDD, the excessive apoptosis of NP cells and production of proinflammatory cytokines and matrix metalloproteinase secretion consist of a pernicious cycle, which leads to the loss of ECM components such as type II collagen and aggrecan [5, 6]. Furthermore, it is known that some proinflammatory factors (IL-1*β* and TNF-*α*) are overly produced in IDD development and drives exacerbation of IDD [7]. Therefore, we constructed an *in vitro* IDD model by treating NP cells with TNF-*α*. Subsequent effects of this research including NP cell apoptosis, matrix metalloproteinase production, and ECM metabolism were investigated in the TNF-*α*-induced IDD cell model.

Retinoid-related orphan receptor alpha (ROR*α*) is a member of the nuclear receptor (ROR) family and was found to mediate multiple biological processes, including cholesterol metabolism, epigenetic regulation, and circadian rhythm [8–10]. ROR*α* was found to effectively bind with cholesterol, act as a ligand-dependent transcriptional factor, and bind to the ROR response element (RORE) [11]. SR3335 was identified as a selective ROR*α* synthetic ligand [12, 13], which acts as an inverse agonist and abolishes the ROR*α*-mediated effect [14, 15]. The study by Choi et al. suggested the critical role of ROR*α* in regulating cartilage matrix metabolism, in which ROR*α* served as the receptor of cholesterol and mediated the damage caused by elevated cholesterol levels [16]. Previous studies found that ROR*α* cooperated with BMAL1 and controlled the activity of HIF-1*α*, thus participating in rhythm regulation in NP cells [17]. However, the exact role of ROR*α* and its inverse agonist SR3335 in the progress of IDD and NP cell degeneration remained unclear.

Yes-associated protein (YAP) is a central regulator of the Hippo pathway with essential functions in cell apoptosis, proliferation, and migration. It has been demonstrated that the activated state of YAP inhibits cell apoptosis [18, 19]. Activated YAP cooperates with transcriptional factor TEAD and regulates transcription of downstream genes, whereas phosphorylated-YAP translocated to the cytoplasm and ensuing proteasomal degradation [20]. Recent studies suggested YAP knockdown accelerated the process of premature senescence of NP cells [21]. Fearing et al. found that NP cell's response to matrix stiffness changes via YAP nuclear translocation [22]. Interestingly, recent studies suggested an intimate relationship between ROR*α* and YAP. Fujita et al. demonstrated that YAP interacted and formed a transcriptional complex with ROR*α* via a specific domain to regulate target gene expression [23]. However, whether modulating ROR*α* via SR3335 affects YAP and further regulates NP cell biological functions remained unclear.

In this study, we demonstrated the anticatabolic and antiapoptosis effect of an inverse agonist of ROR*α*, SR3335, in NP cells. Moreover, we investigated the role between SR3335 treatment and the phosphorylation state of YAP, suggesting that SR3335 may exert such effect in NP cells via regulating the YAP signaling pathway. These findings consistently support the hypothesis that ROR*α* functions as a novel target in IDD by regulating the response of NP cells to hostile environments.

## 2. Materials and Methods

### 2.1. Human NP Tissue Collection

Degenerated lumbar NP tissues were collected from 11 patients (4 males and 7 females; mean age ± SD, 59.2 ± 11.4 years) diagnosed with IDD in magnetic resonance imaging (MRI) and clinical symptoms. In addition, normal NP tissue was collected from 7 patients diagnosed with idiopathic scoliosis (7 females; mean age, 16.7 ± 3.2 years). All patients underwent surgical treatment in the Sun Yat-sen Memorial Hospital of Sun Yat-sen University, and the NP tissues were separated and collected during surgery. The patients signed a formal consent form for collecting tissue samples.

### 2.2. Cell Lines and Cell Culture

Primary human NP cells were purchased from ScienCell (Carlsbad, CA, USA). The NP cells were cultured at 37°C in a 5% CO_2_ incubator with NP cell medium (NPCM, ScienCell). The medium was supplemented with 10% fetal bovine serum (GIBCO, Rockville, MD, USA) and 100 U/ml penicillin and 100 U/ml streptomycin solution.

### 2.3. Animal and IDD Model Construction

Adult Sprague-Dawley rats were purchased from Charles River Laboratories (Beijing, China) and housed in a proper environment and kept in a 12 h light/dark cycle. The rat model of IDD was accomplished with needle puncture of the caudal intervertebral disc as previously described by Qian et al. [24]. Briefly, the section of the caudal intervertebral disc was determined with X-ray. Then, the rat was injected with 3% sodium pentobarbital (30 mg/kg) intraperitoneally. Next, an 18-gauge needle was inserted at the level of the caudal intervertebral disc 5-6. The needle was inserted into the intervertebral disc with a depth of 5 mm and then was rotated 360° before pulling out. The puncture site was disinfected and packed with bandage. Next, the ratswere injected with 10 mg/kg/day SR3335 or DMSO every 3 days for 4 weeks, and then, the rats were sacrificed and subjected to histological analysis.

### 2.4. Antibodies and Reagents

The following antibodies were purchased from CST (Danvers, MA, USA): anticleaved caspase 3, anticaspase 3, anti-YAP, antiphosphorylated-YAP, horseradish peroxidase- (HRP-) conjugated goat anti-rabbit IgG, and goat anti-mouse IgG secondary antibody primary antibodies. Anti-aggrecan, anti-Col2a1, anti-MMP13, and anti-ADAMTS4 primary antibodies were purchased from Abcam (Cambridge, Cambridgeshire, UK). Anti-ROR*α* primary antibody was purchased from Santa Cruz (Santa Cruz, CA, USA). Anti-GAPDH primary antibody was purchased from Proteintech (Houston, TX, USA). SR3335 and Verteporfin were purchased from MedChemExpress (Monmouth, NJ, USA). Recombinant human TNF-*α* was purchased from R&D Systems, Inc. (Minneapolis, MN, USA).

### 2.5. Immunochemical Analysis (IHC)

Nucleus pulposus tissues were washed with PBS and then fixed in 10% paraformaldehyde for 48 h. The tissues were then embedded in paraffin and sectioned at 5 *μ*m. The sections were then deparaffinized, and antigen retrieval was performed by immersing the samples in 95°C EDTA antigen retrieval solution (pH = 6.0, Solarbio) for 30 min. Endogenous peroxidase activity was abolished by 3% hydrogen peroxide treatment for 15 min. Sections were then blocked in 2% normal goat serum for 30 min. Next, sections were incubated with primary antibodies overnight at 4°C. Sections were incubated in HRP-conjugated secondary antibody for 30 min incubation at room temperature. A DAB Horseradish Peroxidase Color Development Kit (ZSGB-BIO) was used for detection. Immunostaining evaluations were performed independently by experimenters blinded to the sample identity. Sections were then immediately washed with tap water, counterstained in hematoxylin for 20 s, and washed again with tap water before dehydration and mounting.

### 2.6. Western Blotting

The NP cells were harvested two days after the indicated treatment and lysed with RIPA lysis buffer (Beyotime, Shanghai, China). The supernatant was collected after centrifugation at 12000 rpm × 10 min at 4°C and was subjected to SDS-PAGE analysis after BCA quantification. Total protein was transferred to nitrocellulose (NC) membranes and then blocked with 5% nonfat milk dissolved in TBST for 1 h. The following antibodies were incubated with the NC membranes in 4°C overnight: anti-GAPDH (1 : 4000), anticleaved caspase 3 (1 : 1000), anticaspase 3 (1 : 1000), anti-BAX (1 : 1000), anti-BCL2 (1 : 1000), anti-YAP (1 : 1000), antiphosphorylated-YAP (1 : 1000), anti-COL2A1 (1 : 1000), anti-MMP13 (1 : 1000), anti-ROR*α* (1:1000), and anti-ADAMTS4 (1 : 1000). The membranes were washed with TBST and then incubated with horseradish peroxidase- (HRP-) conjugated secondary antibodies for 1 h at room temperature. Membranes were visualized using an electrochemiluminescence kit (Millipore). Visualized images were analyzed using the ChemiDoc Imaging System (Bio-Rad, Hercules, CA, USA). Representative images were shown for three replicate experiments. Semiquantitative analyses of the images were conducted using ImageJ.

### 2.7. Real-Time PCR Assay

Total mRNA was extracted using a TRIzol reagent; cDNA was obtained after reverse transcription with a PrimeScript RT Reagent Kit (Novoprotein, Shanghai, China). Real-time PCR was performed with SYBR qRT-PCR SuperMix (Roche, Basel, Switzerland) via a Roche LightCycler 480 System. Relative gene expression was calculated by the 2 − ΔΔCt method. Three biological replicates were performed for each experiment, and the results shown in the figure represent the average ΔCt value of all experiments. The primer sequences used for RT-PCR are listed in Table 1.

### 2.8. Cell Viability Assay

We placed the cell suspension (100 *μ*l/well) in a 96-well plate and incubated in an incubator for 24 hours. Next, 10 *μ*l of CCK-8 solution (MCE) was added to each well. Culture plates were incubated at 37°C for 2 hours. The absorbance at the wavelength of 450 nm was measured using a Sunrise microplate reader (Tecan, Männedorf, Switzerland).

### 2.9. Lentivirus-Mediated Knockdown and Overexpression of RORA Gene

The overexpression vectors of pcDNA3.1-ROR*α*, sh-ROR*α*, and NC-shRNA were designed and synthesized by Genechem (Shanghai, China). The NP cells were seeded in a 6-well plate at a density of 10^5^ cells and then infected with an MOI of 60. After 72 hours, the cells were subjected to fluorescence microscope for infection efficacy analysis. The sequence of anti-ROR*α* short hairpin RNA (sh-ROR*α*) is as follows: 5′-GGAGAAGTCAGCAAAGCAATGCTCGAGCATTGCTTTGCTGACTTCTCC-3′. The sequence of the controlled short hairpin RNA (sh-NC) is as follows: 5′-AAACGTGACACGTTCGGAGAACGAATTCTCCGAACGTGTCACGTTT-3′.

### 2.10. Molecular Docking

The PDB file of ROR*α* (1n83.pdb) was downloaded from the PDB database (https://www.rcsb.org/). The 3D structure file of SR3335 (SR3335.sdf) was downloaded from the NCBI PubChem Compound Database (https://pubchem.ncbi.nlm.nih.gov/). The PDB file was visualized with AutoDock (version 4.2.6). The ligand residues, water molecules, and ions were deleted, and polar hydrogen atoms and Kollman charges were added. After preparing the receptor, ligand file, and related configuration files, we used AutoDock Vina software for docking the ligand molecule into the set receptor pocket. The model and docking pattern were visualized with PyMOL software (version 2.3.4).

### 2.11. Data Analysis

All quantitative data were presented as the mean ± standard deviation (SD). Statistical analysis was performed using one-way analysis of variance (ANOVA) and Student's *t*-test by PRISM 8.0 software (GraphPad, San Diego, California, USA) and SPSS 21.0 software (SPSS, Inc., Chicago, IL, USA). *P* < 0.05 was considered to indicate a statistically significant difference.

## 3. Results

### 3.1. Degenerated NP Tissue Exhibited ROR*α* Upregulation and Increased Apoptosis

**Figure 1:**
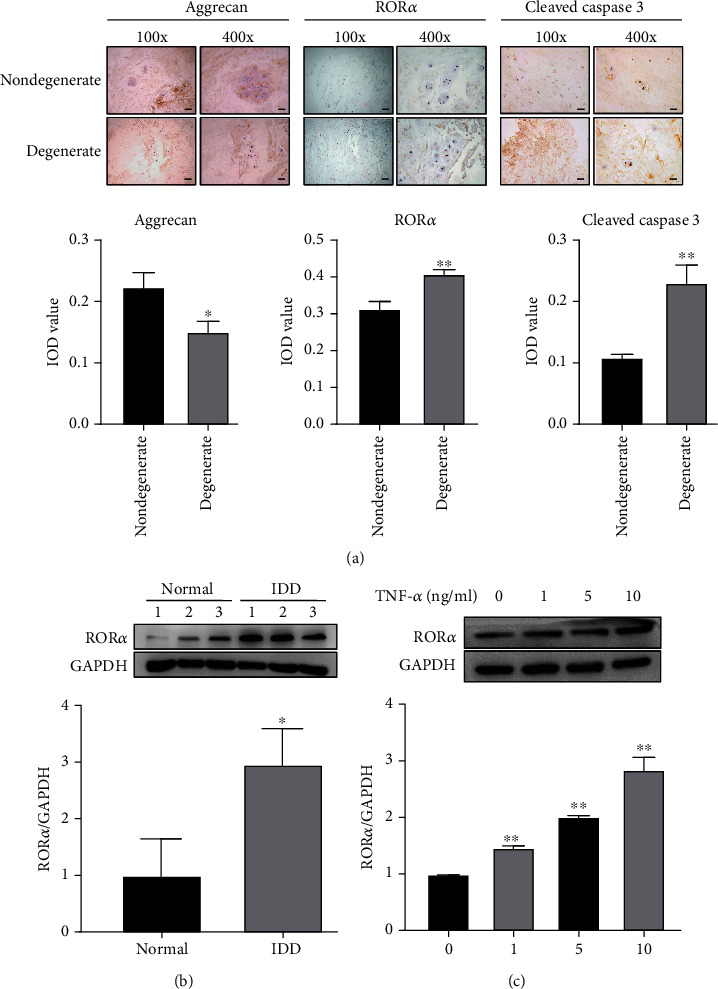
Upregulation of ROR*α* during the process of degeneration of NP tissue. (a) The expression of aggrecan, ROR*α*, and cleaved caspase 3 in normal or degenerated human nucleus pulposus tissue is shown by immunohistochemistry staining. The IOD value for the picture is shown. Scale bar = 200 *μ*m for the 100x image, 50 *μ*m for the 400x image. *N* = 3. (b) Protein levels of ROR*α* in the nucleus pulposus tissue of normal control and IDD patients were determined by western blot analysis. Quantitative analysis of ROR*α* is shown below. ^∗^*p* < 0.05 compared with normal control. (c) Protein levels of ROR*α* in human NP cells treated with the indicated concentration of TNF-*α*. Quantitative analysis is shown below. ^∗∗^*p* < 0.01 compared with the vehicle group. Data are expressed as mean ± SD. All experiments were repeated three times.

Nucleus pulposus tissueswere collected from surgery samples from IDD patients and normal control. Patients who underwent spinal fusion surgery for scoliosis or spine trauma were considered as the normal control. To determine the severity of IDD, we performed immunohistochemistry staining on human nucleus pulposus samples. Our result suggested that the level of aggrecan expression in nucleus pulposus tissue decreased significantly. However, the expression of ROR*α* and ROR*α*-positive cells was elevated in IDD patients, suggesting ROR*α* might have a contributive role in IDD. The expression of an apoptosis marker cleaved caspase 3 in nucleus pulposus tissue was also elevated compared to the normal control tissue (Figure 1(a)). These results suggest that during the pathogenesis of IDD, nucleus pulposus manifest ECM loss, apoptosis, and elevation of ROR*α*. Western blot was applied to determine the expression level of ROR*α* in different patients. Consistent with the IHC staining, ROR*α* was upregulated in the NP tissues of IDD patients (Figure 1(b)). TNF-*α* is an important proinflammatory cytokine that disrupts the biological function of NP cells. Therefore, we sought to find out if TNF-*α* can induce the expression of ROR*α*. After being treated with different concentrations of TNF-*α*, the protein expression level of ROR*α* in NP cells was elevated (Figure 1(c)). These results suggested that ROR*α* was upregulated in both IDD patients or TNF-*α* treated NP cells.

### 3.2. SR3335 Treatment Attenuates Matrix Loss and Apoptosis in the Rat IDD Model

**Figure 2:**
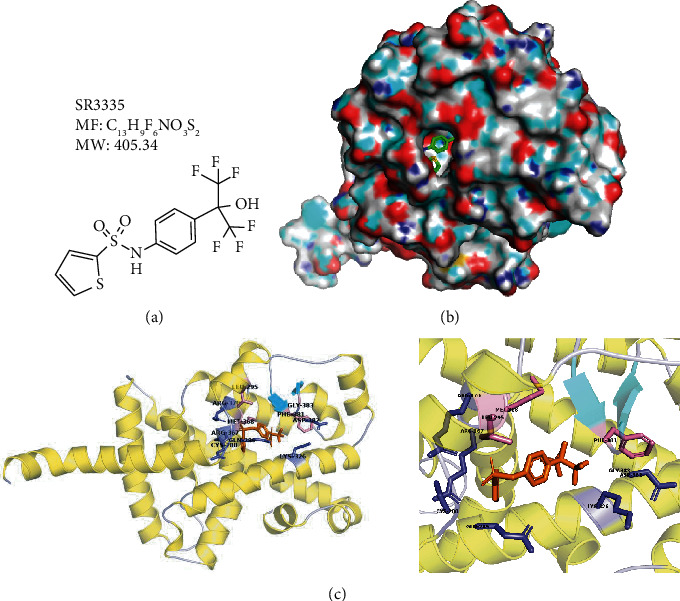
The molecular structure and predicted interaction with the ROR*α* protein. (a) Chemical structure of SR3335. (b) The molecular structure of the ROR*α* protein. The pocket for ligand binding is shown. (c) Molecular docking of SR3335 and ROR*α* indicating high-affinity interaction between SR3335 and adjacent amino acid residue (distance ≤ 2.5 Å).

**Figure 3:**
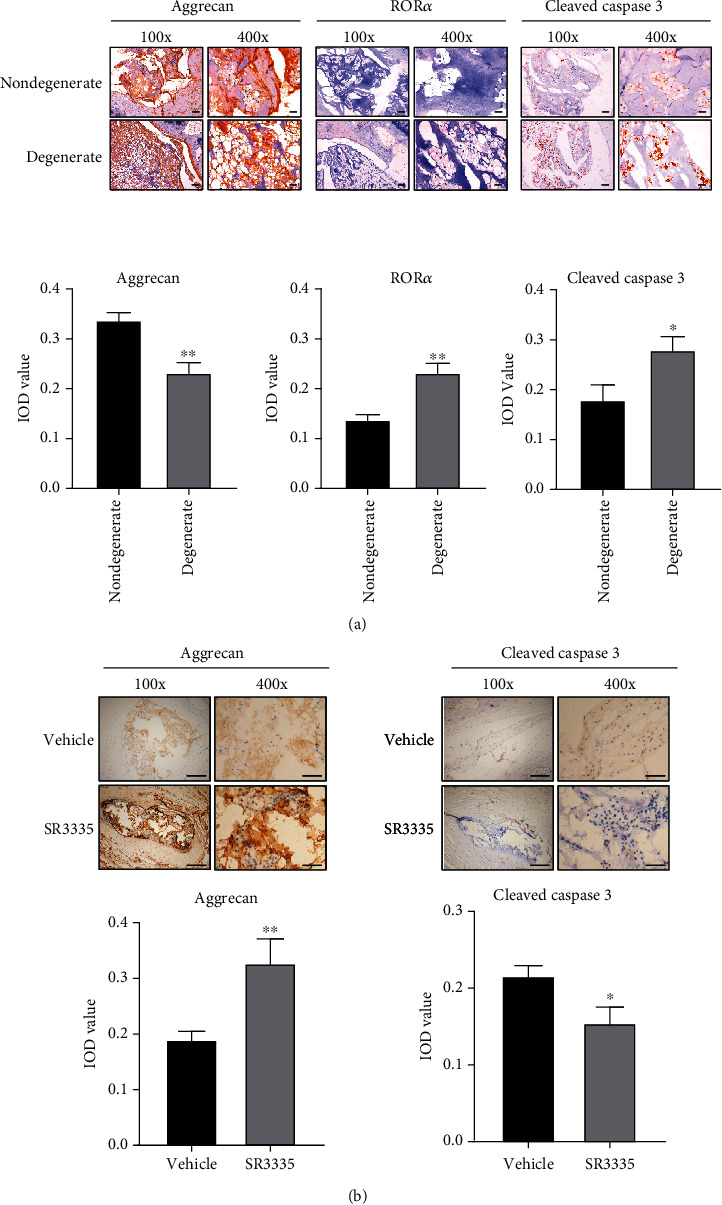
Effect of SR3335 on IDD model rat. (a) The expression of aggrecan, ROR*α*, and cleaved caspase 3 in nucleus pulposus tissue of needle puncture-induced IDD rat model or normal control shown by immunohistochemistry staining. The IOD value for the IHC picture is shown. Scale bar = 200 *μ*m for the 100x image, 50 *μ*m for the 400x image. *N* = 3. (b) The expression of aggrecan and cleaved caspase 3 in nucleus pulposus tissue of needle puncture-induced IDD rat model after SR3335 or vehicle treatment for 4 weeks. The IOD value for the IHC picture is shown. Scale bar = 200 *μ*m for the 100x image, 50 *μ*m for the 400x image.

We next evaluated the effect of ROR*α* inhibition on the apoptosis of NP cells by applicating the inverse agonist SR3335 (Figure 2(a)). We then performed molecular docking on ROR*α* and the inhibitor SR3335. The result suggests high-affinity binding between SR3335 and ROR*α* (Figure 2(b)). To investigate the role of ROR*α* in IDD *in vivo*, we constructed a rat model of IDD by acupuncture of the caudal intervertebral disc. In line with the result in human specimens, the expression of ROR*α* and cleaved caspase 3 was elevated, while the expression of aggrecan decreased in the IDD group (Figure 3(a)). After constructing the IDD model, the rats were injected with 10 mg/kg/day SR3335 or DMSO for 4 weeks. Severe aggrecan loss was observed in the DMSO-treated group, while SR3335 treatment restored the expression of aggrecan. Besides, SR3335-treated rats exhibited lower apoptosis as was indicated by cleaved caspase 3 (Figure 3(a)).

### 3.3. SR3335 Treatment Attenuates TNF-*α*-Induced Apoptosis in Human NP Cells

**Figure 4:**
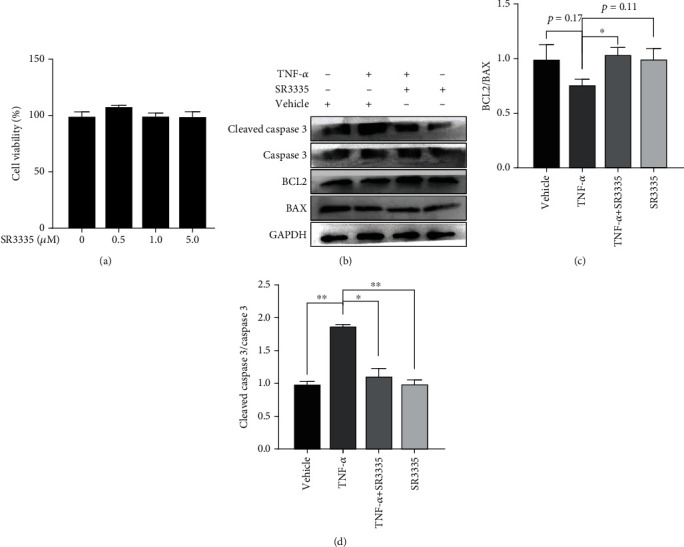
Effect of SR3335 on NP cell viability and apoptosis. (a) NP cells were treated with the indicated concentration of SR3335 (0.5, 1.0, and 5.0 *μ*M), and the cell viability was measured with the CCK-8 assay. *N* = 3. (b) The NP cells were pretreated with 1 *μ*M of SR3335 2 h before 10 ng/ml TNF-*α* treatment. The protein level of cleaved caspase 3, caspase 3, BCL2, and BAX was measured by the western blot. (c) Quantification of BCL2 and BAX. The ratio of BCL2/BAX was measured. ^∗^*p* < 0.05 compared with the TNF-*α* group. (d) Quantification of cleaved caspase 3 and caspase 3. The ratio of cleaved caspase 3/caspase 3 was measured. ^∗^*p* < 0.05 or ^∗∗^*p* < 0.01 compared with the TNF-*α* group. Data are expressed as mean ± SD. All experiments were repeated three times.

To determine whether SR3335 affects the proliferation of NP cells, a CCK-8 assay was performed. Treatment with SR3335 did not affect the viability of NP cells (Figure 4(a)). Western blot was performed to detect the expression of key regulators in apoptosis pathways, including cleaved caspase 3, BCL2, and BAX. The ratio of BCL2/BAX and cleaved caspase 3/caspase 3 was quantified. These results showed that NP cells treated with TNF-*α* express higher cleaved caspase 3/caspase 3 ratio and lower BCL2/BAX ratios, suggesting increased apoptosis in the TNF-*α* treated group. However, SR3335 treatment reversed the imbalance of BCL2/BAX and cleaved caspase 3/caspase 3 ratios, suggesting SR3335 treatments prevent the apoptosis of NP cells (Figures 4(b)–4(d)). These results indicate that SR3335 does not affect the viability of NP cell but reverses the increased apoptosis induced by TNF-*α*.

### 3.4. SR3335 Protects Human NP Cells from the Procatabolic and Antianabolic Effect Induced by TNF-*α*

**Figure 5:**
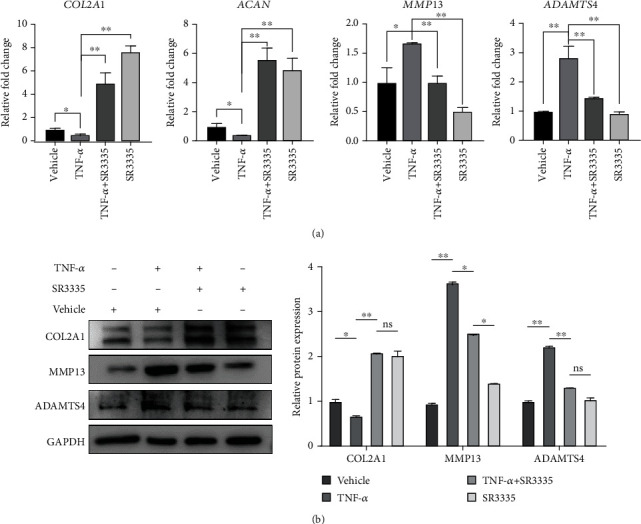
SR3335 treatment decreased the nucleus pulposus ECM catabolism upon TNF-*α* treatment. The NP cells were pretreated with 1 *μ*M of SR3335 2 h before 10 ng/ml TNF-*α* treatment. (a) The mRNA level of COL2A1, ACAN, MMP13, and ADAMTS4 was assayed by real-time PCR. Data are expressed as mean ± SD. ^∗^*p* < 0.05, ^∗∗^*p* < 0.01, and ^∗∗∗^*p* < 0.001 compared with the TNF-*α* treated group. (b) The protein level of COL2A1, MMP13, and ADAMTS4 was assayed by western blot and quantification.

**Figure 6:**
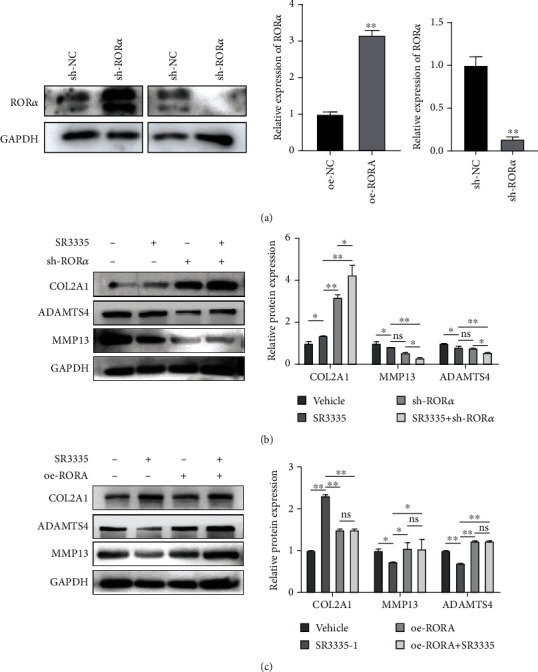
The anticatabolism and proanabolism effect of SR3335 is mediated by interaction with ROR*α*. (a) The expression of ROR*α* protein after being transfected with RORA overexpression or sh-RORA lentivirus was measured with western blot. (b) The NP cells were transfected with sh-RORA lentivirus and 1 *μ*M of SR3335. The protein level of COL2A1, MMP13, and ADAMTS4 was assayed by western blot and quantification. (c) The NP cells were transfected with RORA overexpression lentivirus and 1 *μ*M of SR3335. The protein level of COL2A1, MMP13, and ADAMTS4 was assayed by western blot and quantification. All experiments were repeated three times.

In order to investigate the protective role of ROR*α* inverse agonist SR3335 in regulating human NP cell metabolism, we used TNF-*α*) as a stimulatory factor for inflammatory environment. We used qRT-PCR to measure the expression level of the NP cell metabolic marker. The expression level of matrix catabolic markers MMP13 and ADAMTS4 was elevated upon TNF-*α* treatment, while the mRNA expression of nucleus pulposus ECM component genes COL2A1 and ACAN was decreased. Treatment with SR3335 restored the decrease of COL2A1 and ACAN (*p* < 0.01) and suppressed the increased expression of MMP13 and ADAMTS4 (Figure 5(a)). We subsequently evaluated the protein level of type II collagen, MMP13, and ADAMTS4, and the result correlates with the result of qRT-PCR (Figure 5(b)). We next constructed sh-ROR*α* or oe-ROR*α* lentivirus to investigate whether RORA overexpression or knockdown affected the procatabolic and antianabolic effects on nucleus pulposus cells. The efficiency of knockdown or overexpression was assessed with western blot (Figure 6(a)). We found that ROR*α* knockdown can synergize with SR3335 to inhibit the expression of MMP13 and ADAMTS4 (Figure 6(b)). However, ROR*α* overexpression partially reversed the effect of the SR3335 treatment (Figure 6(c)). These results suggest that SR3335 reversed the dysregulation induced by TNF-*α* and such effect is mediated via interaction with ROR*α*.

### 3.5. SR3335 Mediated the Protective Effect in NP Cells by Regulating the Phosphorylation State of YAP

**Figure 7:**
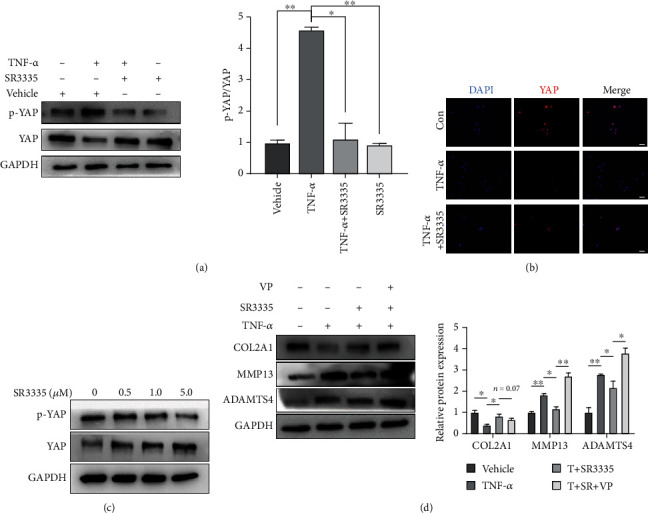
ROR*α* inhibition suppressed the phosphorylation and inactivation of YAP induced by TNF-*α*. (a) The expression levels of YAP and p-YAP were measured by western blot and quantification of p-YAP and YAP. The ratio of p-YAP/YAP was calculated. Data are expressed as mean ± SD. ^∗^*p* < 0.05 and ^∗∗^*p* < 0.01 compared with the TNF-*α*-treated group. (b) Immunofluorescence staining of YAP showed the location and relative fluorescence strength under vehicle, TNF-*α*, TNF-*α*+SR3335 stimulation. Scale bar = 50 *μ*m. (c) NP cells were treated with various dosages of SR3335 (0, 0.5, 1.0, and 5.0 *μ*M) for 2 hours. The levels of p-YAP and YAP were assayed by western blot. (d) NP cells were treated with 10 ng/ml TNF-*α*, 1 *μ*M SR3335, or 1 *μ*M VP for 24 h. The protein level of COL2A1, MMP13, and ADAMTS4 was assayed by western blot and quantification. All experiments were repeated three times.

Recently, increasing evidence suggested that the Hippo signaling pathway plays a crucial role in regulating degenerative disc diseases [21]. Therefore, we next investigate whether the important downstream regulator of the Hippo signaling pathway, YAP, is regulated by SR3335. YAP was inactivated and relocated to the cytoplasm when the protein was phosphorylated at S127, and subsequently underwent ubiquitin-mediated protein degradation. Under 10 ng/ml TNF-*α* treatment, YAP protein was phosphorylated and the p-YAP/YAP ratio was elevated significantly. However, SR3335 treatment restored the elevation of the p-YAP/YAP ratio (Figures 7(a) and 7(b)). Moreover, SR3335 treatment inhibited the phosphorylation of YAP in a dose-dependent manner (Figure 7(c)). To evaluate whether SR3335 affected NP cells in a YAP-dependent way, we used a well-established YAP inhibitor Verteporfin (VP) to disturb the interaction between YAP and TEAD. The result showed that VP treatment reversed the anticatabolism effect of SR3335, characterized by upregulating MMP13 and ADAMTS4 expression. The expression of COL2A1 was downregulated upon VP treatment (Figure 7(d)). These results suggest that SR3335 regulates YAP's phosphorylation state and protein level and mediates the protective efforts on NP cells through the Hippo/YAP signaling pathway.

## 4. Discussion

ROR*α* is a member of the nuclear receptor family, which acts as a ligand-dependent transcriptional factor that participates in multiple biological processes in the musculoskeletal system, including osteoblast differentiation, chondrocyte hypertrophy, and skeletal muscle homeostasis [25–27]. Here in the present study, we sought to investigate the role of ROR*α* in NP cells and the pathogenesis of IDD. The expression level of ROR*α* was elevated in both clinical IDD patients and the puncture-induced IDD rat model. ROR*α* inverse agonist SR3335 suppressed TNF-*α*-induced elevation of matrix catabolism enzymes MMP13 and ADAMTS4 while restoring the decline of the matrix hallmark molecular type II collagen and aggrecan. Besides, SR3335 attenuates the increased apoptosis rate in TNF-*α* treated NP cells. *In vivo* investigation suggested that intraperitoneal injection of SR3335 reversed the dysregulation of aggrecan and cleaved caspase 3. Moreover, ROR*α* exerted such effect partly through the Hippo/YAP signaling pathway. In conclusion, these results indicated that ROR*α* protects NP cells from the TNF-*α*-induced matrix catabolism and apoptosis via regulating YAP.

NP cells have been considered the only cell type in nucleus pulposus, composed of mucoid-like extracellular matrix (ECM) rich in type II collagen and aggrecan. During the process of IDD, a significant loss of NP cells was observed, and this pathological change may be attributed to the increased rate of apoptosis. Therefore, the increased rate of apoptosis leads to a decrease in secretion of ECM. In the process of ECM secretion dysfunction, the expression level of type II collagen, aggrecan, MMPs, and ADAMTSs changed significantly, and the pattern can therefore be used as biomarkers of nucleus pulposus degeneration [28, 29]. This study discovered that the inhibition of ROR*α* by SR3335 reversed the dysregulation of ECM markers in TNF-*α* treated NP cells.

ROR*α*, a crucial receptor in the cholesterol pathway, is now regarded as an important and promising target in treating many diseases, including nonalcoholic steatohepatitis and cardiac hypertrophy [15, 30]. The role of cholesterol in the pathogenesis of various degenerative orthopedic disorders has been studied recently [31]. The study by Zhang et al. suggests the lipid dysregulation correlates with a higher risk of lumbar disc herniation [32]. Intradiscal injection of simvastatin may reverse collagen type II loss in IDD model rats [33]. Genetic ablation of apolipoprotein E (ApoE) in rodents and rabbits has been used as a promising animal model of lipid metabolism and atherosclerosis. Interestingly, APOE-knockout rabbit exhibits susceptibility to premature intervertebral disc degeneration [34]. Therefore, targeting the cholesterol metabolism pathway might be a strategy in treating IDD. In the study we presented here, the inverse agonist of ROR*α*, SR3335, protected NP cells from the hostile microenvironment exerted by TNF-*α*. The effects are possibly mediated through the regulating matrix metabolism and NP cell apoptosis. ROR*α* plays a complex role in regulating apoptosis in different cell types. Loss of ROR*α* results in progressive, diverse testicular damage and leads to apoptosis, suggesting that ROR*α* knockout promotes apoptosis in the testicle [35]. However, other studies revealed the proapoptosis effect of ROR*α* in different cell types [36, 37], suggesting the complexity of ROR*α* in regulating cell senescence and apoptosis.

TNF-*α* is regarded as one of the most critical factors contributing to IDD's pathogenesis [38, 39]. During intervertebral disc degeneration, increased expression of TNF-*α* was observed. The level of TNF-*α* correlates with the severity of degeneration [40]. The biological effects of TNF-*α* were mainly mediated through the TNF receptor and subsequently regulates the transcriptional activity of downstream genes. With the elevation of TNF-*α*, increased apoptosis, senescence, and matrix degradation enzyme secretion were observed in the NP cells. The loss of ECM eventually leads to the narrowing of the intervertebral space and causes the symptom of low back pain. Therefore, TNF-*α* treatment has been used as a classic model for mimicking the inflammation environment in IDD [41]. In this study, we found ROR*α* was elevated in degenerated NP tissue, and TNF-*α* can induce the up-regulation of ROR*α*. We thus speculated that ROR*α* is a downstream effector for TNF-*α* and ROR*α* blockade may eliminate the negative effect of TNF-*α*.

The Hippo/YAP signaling pathway is important in multiple biological processes, including organogenesis, mechanical stress transduction, and degenerative diseases. Deng et al. demonstrated that inflammatory cytokines trigger the degradation of YAP and lead to subsequent matrix-degrading enzyme secretion, which exacerbates the pathologic feature of osteoarthritis [42]. Furthermore, overexpression of YAP attenuates the PMMA-induced intervertebral disc degeneration in rats [43]. YAP also mediates the downstream effect of inflammatory cytokines, such as interleukin-6, to promote epithelial regeneration [44]. In this study, we found that TNF-*α* induced the phosphorylation of YAP and decreased the expression of unphosphorylated-YAP in NP cells. Moreover, inhibition with inhibitor VP-disrupted YAP activation leads to elevation of MMP13 and ADAMTS4, which affirmed the protective role of YAP under inflammatory status.

## 5. Conclusion

In summary, we focus on the expression of ROR*α* and its well-established inverse agonist SR3335 during NP cell degeneration. SR3335 treatment reversed the loss of matrix components in the puncture-induced rat IDD model. *In vitro* studies suggested that SR3335 treatment reversed the dysregulation of COL2A1, MMP13, and ADAMTS4. Besides, SR3335 also reversed the increased apoptosis of NP cells, and such effects are mediated through the Hippo/YAP pathway. Our findings reveal the potential of SR3335 in treating intervertebral disc degeneration.

## Figures and Tables

**Table 1:** Primers used for real-time quantitative PCR.

Gene	Primer	Sequence (5′-3′)
*GAPDH*	Forward	AGAAAAACCTGCCAAATATGATGAC
	Reverse	TGGGTGTCGCTGTTGAAGTC

*COL2A1*	Forward	GGCAATAGCAGGTTCACGTACA
	Reverse	CGATAACAGTCTTGCCCCACTT

*ACAN*	Forward	TGCATTCCACGAAGCTAACCTT
	Reverse	GACGCCTCGCCTTCTTGAA

*MMP13*	Forward	GAGGAGGAGATCGTGTTTCCA
	Reverse	CCAGCTCTAGTAGCAGCGTC

*ADAMTS4*	Forward	CCAGACTTCACGATGGCATTG
	Reverse	GGCATCTCCTCCATAATTTGGC

GAPDH: glyceraldehyde-3-phosphate dehydrogenase; COL2A1: collagen type II; ACAN: aggrecan; MMP13: matrix metalloproteinase 13; ADAMTS4: a disintegrin and metalloproteinase with thrombospondin motifs 4.

## Data Availability

The data used to support the findings of this study are included within the article.
